# Translation and validation of the Brown attention-deficit disorder scale for use in Brazil: identifying cases of attention-deficit/hyperactivity disorder among samples of substance users and non-users. Cross-cultural validation study

**DOI:** 10.1590/1516-3180.2017.0227121217

**Published:** 2018-04-16

**Authors:** Simone Mayumi Kakubo, Mariel Mendez, Juliana Doering Silveira, Leonardo Maringolo, Conrado Nitta, Dartiu Xavier da Silveira, Thiago Marques Fidalgo

**Affiliations:** I Medical Student, Universidade Federal de São Paulo (UNIFESP), São Paulo (SP), Brazil.; II MPH. Public Health Professional, Mailman School of Public Health, Columbia University, New York, United States.; III MD. Attending Physician, Department of Psychiatry, Universidade Federal de São Paulo (UNIFESP), São Paulo (SP), Brazil.; IV MD. Attending Physician, Department of Psychiatry, Universidade Federal de São Paulo (UNIFESP), São Paulo (SP), Brazil.; V MD. Attending Physician, Department of Psychiatry, Universidade Federal de São Paulo (UNIFESP), São Paulo (SP), Brazil.; VI MD, PhD. Full Professor, Department of Psychiatry, Universidade Federal de São Paulo (UNIFESP), São Paulo (SP), Brazil.; VII MD, PhD. Affilliate Professor, Department of Psychiatry, Universidade Federal de São Paulo (UNIFESP), São Paulo (SP), Brazil.

**Keywords:** Attention deficit disorder with hyperactivity, Mental disorders, Substance-related disorders, Psychiatry, Comorbidity

## Abstract

**BACKGROUND:**

The Brown Attention-Deficit Disorder Scale (BADDS) was developed as a self-report assessment that was designed to screen for presence of symptoms of attention deficit hyperactivity disorder (ADHD). The objective here was to translate and validate the adult self-report BADDS for use in Brazil.

**DESIGN AND SETTING:**

Cross-cultural validation study conducted in an addiction unit at a public university hospital.

**METHODS:**

This study included a control group (n = 100) and a drug-user group (n = 100). Both groups included subjects aged 18 to 60 years old. The control group had no prior diagnosis of drug addiction and the drug-user group included participants with a diagnosis of addiction. Each participant answered Brazilian Portuguese translations of both the BADDS and the Adult Attention Deficit Hyperactivity Disorder Self-Report Scale (ASRS) questionnaires, in paper-and-pencil format.

**RESULTS:**

The drug-user group scored higher than the control group on both scales. The mean scores on ASRS were 27.26 (standard deviation, SD: 11.99) and 25.85 (SD: 8.65) respectively (P > 0.05). The mean scores on BADDS were 79.56 (SD: 29.61) and 79.31 (SD: 18.09), respectively (P > 0.05). Cronbach’s alpha for BADDS was 0.95. BADDS presented fair sensitivity (72% accuracy) and fair specificity (88% accuracy).

**CONCLUSION:**

This study provides discriminative validity evidence for use of BADDS among Brazilian adults with substance-use disorders.

## INTRODUCTION

Until recently, it was believed that attention deficit hyperactivity disorder (ADHD) was exclusively a pediatric condition.^1^ However, current research indicates that 60% to 70% of children diagnosed with ADHD continue to manifest symptoms into adulthood.^2^ Persistence of symptoms of ADHD can have a pressing impact on the safety and personal relationships of patients, as well as having secondary effects in adulthood such as lost days of productivity and continual negative feedback or social and educational disadvantages.^3^ A recent study that used the Diagnostic and Statistical Manual of Mental Disorders-IV (DSM-IV) criteria for ADHD, which was conducted in both developed and underdeveloped countries, estimated that the worldwide prevalence of ADHD was 3.4% and showed that it was higher among underdeveloped countries.^4^

Currently, there are no biomarkers available for diagnosing ADHD. All diagnoses require careful assessments by clinicians through interviews and appropriate classification criteria.^5^ Two diagnostic tools are used today to classify this disorder: DSM-5 and the International Statistical Classification of Diseases and Related Health Problems (ICD-10).

These two diagnostic tools define ADHD as a hyperkinetic disorder, a disorder characterized by inattention, hyperactivity and impulsivity with onset in childhood or adolescence. It is believed that the current diagnostic criteria (both DSM-5 and the ICD-10) are inadequate for evaluation of adults because they focus on early childhood problems and they do not fully account for developmental and maturation changes.^6^ The symptoms and functional impairments identified among adults for making a diagnosis of ADHD tend to be different from those observed among children. In cases of diagnosing children, parents and teachers play a key role in recognizing, identifying and rating the child, based on standardized evaluation scales.^7^ On the other hand, for adults, there is usually no one who has observed symptoms or problems with their behavior. Therefore, the diagnosis of the disorder is based upon a self-report of behaviors. Research has indicated that adolescents and adults with ADHD often underestimate their symptoms,^8^ thus making diagnosis much more difficult.

Many people who suffer from ADHD may also be at risk of having co-occurring psychiatric disorders or chronic illnesses. It has been estimated that more than 87% of adults with ADHD have some form of comorbidity.^9^ A study conducted in the United States in 2008 demonstrated that adults with ADHD had comorbidities involving anxiety (47%), mood disorders (38%), impulse control (20%) and substance-use disorders (SUD) (15%).^10^ The prevalence of having a comorbidity involving substance use is significantly higher among individuals with ADHD than among those without ADHD.^11^ It has been shown that adults with substance-use disorders are at higher risk of presenting ADHD and earlier onset of ADHD, with greater severity of SUD and chronic SUD.^12^ Furthermore, ADHD has been linked to lower remission rates for cigarette smoking and SUD.^12^ Since the impact of comorbidity between ADHD and SUD in adulthood is significant, earlier diagnosis, treatment and healthcare delivery are relevant for patient prognosis.^12^ This highlights the importance of studying this specific population of substance users with ADHD as a comorbidity.

In order to enhance and assist the diagnostic process for some psychiatric conditions, standardized instruments are becoming increasingly necessary. Standardized assessment instruments are widely disseminated within research and have increasingly been used as a resource for evaluating different aspects of mental health. In clinical practice, standardized instruments are critical for screening and diagnosing patients. Currently, researchers use self-report questionnaires as a critical part of screening and diagnosing patients with ADHD. Limitations exist because the screening and diagnosis tool is unavailable in other countries.

To improve the reach of the Brown Attention-Deficit Disorder Scale (BADDS) in Brazil, the aim of this study was to translate and validate it for use in Portuguese among a Brazilian sample of drug users and among a sample of people with no history of drug use.

## METHOD

### Study design, setting and ethics

This was a translation and cross-cultural validation study, conducted at the Federal University of São Paulo (Universidade Federal de São Paulo, UNIFESP). The ethics committee of UNIFESP approved the study (June 7, 2013; no. 17280313.2.1001.5505) All participants signed an informed consent statement.

### Questionnaire translation

BADDS is a self-report questionnaire that is used for screening adults with a possible case of ADHD.^13^ Differently from other scales like ASRS, BADDS does not contain any DSM-5 criteria. The questions in BADDS are not driven in terms of inattention-hyperactivity-impulsivity symptoms, but instead assess functional impairment in five areas, through 40 questions. These five areas are as follows:

1. organizing and prioritizing work and activation for work;
2. focusing on tasks, sustaining this focus and shifting attention to tasks;
3. regulating alertness and sustaining effort, and the ensuing processing speed;
4. managing frustration and modulating emotions; and
5. using working memory and accessing recall.

Each question has a possible score from 1 to 4. The higher the cluster score and overall score are, the higher the risk is that the individual has ADHD.

All individuals who complete the BADDS questionnaire are classified into three groups: i) possible, but unlikely to have ADHD, if the score is less than 40; ii) possible, but unconfirmed ADHD, if the score is between 40 and 54; and iii) highly likely but unconfirmed ADHD, if the score is above 55.

A 2008 study demonstrated that BADDS was more reliable than were other instruments that were based on the DSM-IV criteria.^14^ The information provided by the patient via this self-report questionnaire and through information from someone close to the patient is more accurate for assessing ADHD symptoms than is use of the DMS-5 criteria.

The paper-and-pencil format of BADDS was translated into Portuguese. The translation was conducted using a two-step procedure, known as the back-translation method, as recommended by Brislin (1973) and by Smit (2006).^15^^,^^16^ According to these authors, two bilingual translators are required in order to come to a consensus regarding any translational difficulties or discrepancies.^15^^,^^16^ In our case, a bilingual psychiatrist first translated the items from English to Portuguese, followed by back-translation into English conducted by a linguist. The discrepancies between the two versions were resolved by reaching a consensus between the two bilingual professionals.

ASRS is currently the most accepted and most widely used self-report questionnaire for screening for ADHD symptoms.^17^ The questionnaire asks directly about the existence of inattention-hyperactivity-impulsivity symptoms, in the way in which these are presented in the DSM-5 criteria. It consists of 18 questions, with scores for each question ranging from 0 to 4. Zero means that no symptoms were present within the last six months, while 4 indicates that all symptoms were present within the last six months. The composite scores of this questionnaire, similarly to BADDS, classifies patients into categories depending on the risk of ADHD. Patients with scores between 0 and 16 are considered to be individuals with an unlikely risk of having ADHD; patients with scores between 17 and 23 are considered to be individuals with a likely chance of having ADHD; and finally, individuals with scores of 24 and over are considered to present a high likelihood of having ADHD. 

ASRS was chosen as the gold standard for this study. The main reason for this choice was that the Addiction Unit did not have enough trained psychiatrists to perform a complete ADHD diagnosis on these 100 subjects. It is important to state that this unit is not specifically designed for research purposes and that this evaluation would imply a significant increase in the psychiatrists’ workload. 

### Participants

The validation study sample consisted of two groups. One group (control group) consisted of a convenience sample of 100 students from UNIFESP, in accordance with the following inclusion criteria: i) between the ages of 18 and 60 years; ii) either female or male; iii) literate, independent of education level, socioeconomic level or ethnicity; and iv) no prior diagnosis of psychiatric conditions or drug addiction (according to self-report).

The second group (drug users) comprised 100 adults who were currently attending an outpatient facility, the Addiction Unit of UNIFESP (through the Guidance and Attendance Program for Substance Dependents; Programa de Orientação e Atendimento a Dependentes, PROAD). At this facility, patients participate in weekly group therapy sessions and are individually evaluated by a psychiatrist at least once a month. This trained psychiatrist is responsible for making the diagnosis of drug dependence, using the DSM-5 criteria. Individual sessions with a psychologist may form part of the treatment, depending on the needs of each patient. For our second group, the same inclusion criteria were used, with the addition that all patients had a diagnosis of substance dependence, which had been assessed and diagnosed by a psychiatrist in accordance with the DSM-5 criteria. All substances except tobacco were included in the assessment.

All participants in both groups answered two self-report questionnaires: BADDS and the Adult ADHD Self-Report Scale (ASRS). Aside from age, gender and psychiatric diagnosis, no other sociodemographic or clinical information was collected.

### Statistical analysis

The total scores on both scales were tested in order to check for normal distribution, which was confirmed. Chi-square tests were used to analyze categorical data, while t tests were used to analyze parametric continuous variables. The internal consistency of BADDS was measured by means of the Cronbach’s alpha method. Alpha was computed by correlating the score for each scale item with the total score for each individual observation, and then making comparisons with the variance for all individual item scores.^18^

The participants’ scores were compared using the means that were obtained through the two questionnaires, to determine criteria for concurrent and discriminant validation measurements for BADDS. Given the scores from each questionnaire, several cutoff points were verified for sensitivity and specificity, in increments of 10 (instead of the usual one-by-one increments of scores that are used for most of the instruments available).

To analyze cutoff points, the receiver operating characteristic (ROC) curve was used. The statistical significance level was taken to be 0.05. The statistical analysis software Statistical Package for the Social Sciences (SPSS 22.0) for Windows was used.

## RESULTS

Within the drug-user group, men comprised 87% of the sample, whereas in the control group, men represented 47% of the total sample. The drug-user group scored higher than the control group in both ADHD instruments. The mean score from the ASRS questionnaire in the drug-user group was 27.26 (SD: 11.99), compared with 25.85 (SD: 8.65) in the control group (P > 0.05). The mean scores from the BADDS questionnaire were 79.56 (SD: 29.61) and 79.31 (SD: 18.09), respectively (P > 0.05). Cronbach’s alpha from BADDS was 0.95.

**Figure 1:**
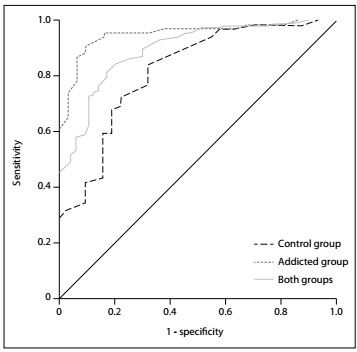
Receiver operating characteristic curve of the control group (n = 100), addicted group (n = 100) and both groups together (n = 200).

Table 1 summarizes the results from the ROC analysis (Figure 1). The optimum cutoff score was 50, as shown in Table 1. Scores below 50 indicate a negative diagnosis for ADHD, whereas scores of 51 and over indicate a positive diagnosis. Using this threshold, the substance abuse scale detected true positives (sensitivity) with 72% accuracy and true negatives (specificity) with 88% accuracy. For both groups, the area under the curve was 0.891.

**Table 1:** Cutoff points compared between control group, addiction unit group (treated at PROAD) and both groups together (total)

Cutoff^**a**^	Total^**b**^		PROAD^**c**^		Control group^**d**^	
	Sensitivity	Specificity	Sensitivity	Specificity	Sensitivity	Specificity
10	1	0	1	0	1	0
20	0.985	0.225	0.985	0.322	0.985	0.129
30	0.971	0.451	0.971	0.516	0.971	0.387
40	0.869	0.693	0.956	0.709	0.782	0.677
50	0.724	0.887	0.869	0.935	0.579	0.838
60	0.492	0.951	0.652	0.967	0.333	0.935
70	0.318	1	0.478	1	0.159	1
80	0.246	1	0.391	1	0.101	1
90	0.137	1	0.217	1	0.057	1
100	0.050	1	0.101	1	0	1
110	0.028	1	0.057	1	0	1
120	0.014	1	0.028	1	0	1
> 120	0	1	0	1	0	1

^a^Cutoff points as presented in the method section; ^b^Total = scores of the PROAD and control groups together; ^c^PROAD = scores of the addiction unit group; ^d^Control group = scores of the control group.

## DISCUSSION

This study demonstrated that BADDS, a tool for diagnosing ADHD among adults, has high internal consistency and differentiates possible cases of ADHD among people with concurrent substance use and people without a psychiatric diagnosis. The scale has fair sensitivity and specificity.

These results encourage use of BADDS for identifying possible ADHD cases, both in clinical practice and in research. Screening for ADHD using this scale enables greater agility in reaching diagnostic confirmation of ADHD. Moreover, this tool results in the following:

1. higher quality for the service, given that a standard is created for diagnostic investigation;
2. improvement of adherence to treatment, since patients can track their progress through reductions in the scores; and
3. greater focus of the available resources on individuals who are accurately screened positive, so that they can be evaluated by a psychiatrist or a psychologist.

In this context, it is essential to develop and validate these important tools, not only to improve the diagnostic process, but also to allocate resources where needed.

Once validated, the BADDS scale can become a screening tool that could improve psychiatric care and provide resources for those who truly are ADHD-positive. The self-report format, the non-medical language and the fact that the questions do not examine only the presence of symptoms make the Brown Attention-Deficit Disorder Scale a valuable instrument for use by any healthcare professional who wants to optimize ADHD healthcare services (Annex 1).

Diagnosing psychiatric disorders is complex, and this is even more so when they are associated with other psychiatric comorbidities, as is the case with ADHD. The lack of biomarkers to identify psychiatric conditions makes validation tools necessary, to minimize diagnostic difficulties.

Screening instruments are useful and increase the quality of healthcare services. Validated screening tools provide cost-effectiveness strategies, reinforce diagnostic accuracy and allow exploration of different aspects of mental health. In addition, they adds to the body of knowledge of overall mental health examination and care.

Since the demand for mental health facilities is greater than the number of care services available in Brazil, validation of the BADDS questionnaire provides the possibility of extrapolating the sphere of psychiatric consultation offices and could be a way to reduce the gap in mental health facilities. It could reduce the burden on mental health facilities, while simultaneously correctly identifying cases of adults living with ADHD.

### Limitations

Some limitations of the present study need to be noted. Because a self-report questionnaire was used, rather than psychiatric interviews, the questions were subject to interpretation by the participants and to possible information bias. Moreover, a psychiatric interview should be the gold standard, but this was not possible because of limitations to the capacity of our facility. In addition, this was a cross-sectional survey and therefore associations do not imply causation. Lastly, although sensitivity and specificity are characteristics of each test, positive and negative predictive values depend on the prevalence of the condition studied within a given sample. Therefore, in populations with low prevalence of ADHD, the positive predictive value tends to be low and the negative predictive values tend to be high. This means that, although the findings through an instrument might rule out a diagnosis of ADHD, there is a high chance that any positive results will in fact be false positives.

## CONCLUSION

In summary, this study conducted on a Brazilian sample demonstrated that BADDS has discriminative validity for making diagnoses of ADHD. The ROC curve analyses showed the usefulness of BADDS for detecting adults who need ADHD treatment, particularly among those with substance-use disorders.

## Figures and Tables

**Annex 1:**
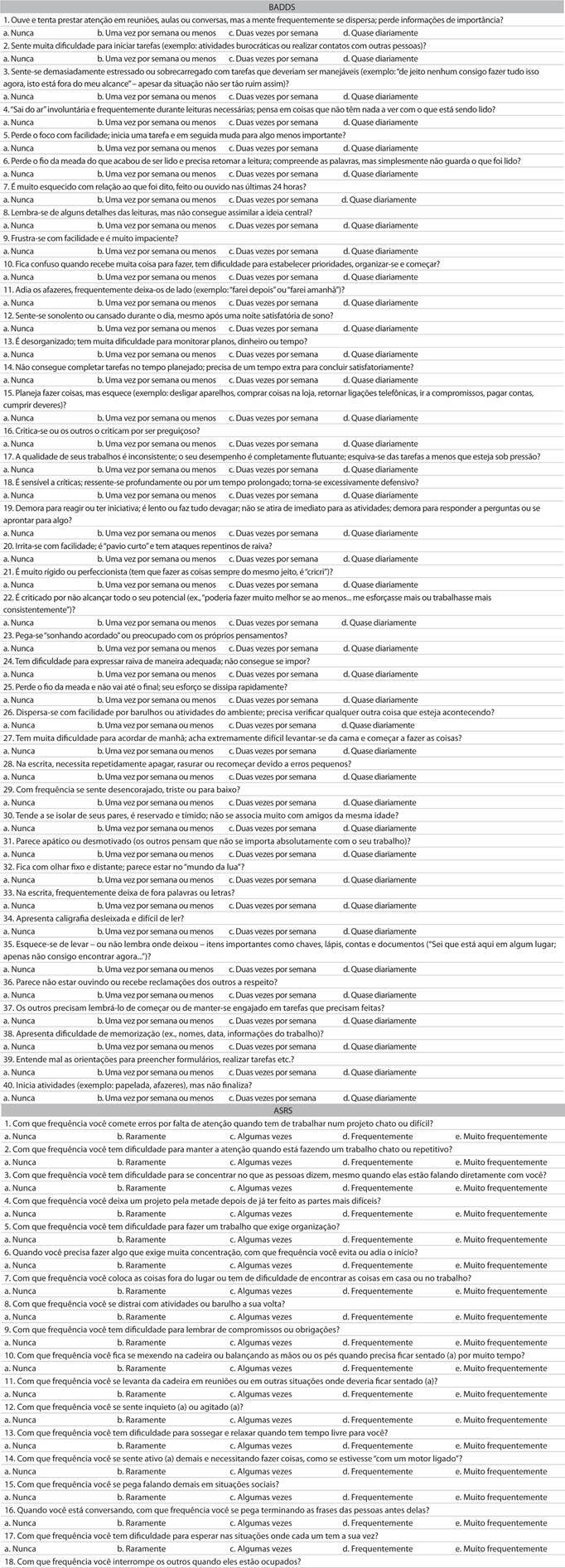
Brown Attention-Deficit Disorder Scale (BADDS) and Adult Self-Report Scale (ASRS) for ADHD
